# Comparing Antimicrobial Susceptibilities among *Mycoplasma pneumoniae* Isolates from Pediatric Patients in Japan between Two Recent Epidemic Periods

**DOI:** 10.1128/AAC.02517-18

**Published:** 2019-06-24

**Authors:** Tomohiro Oishi, Kento Takahashi, Shoko Wakabayashi, Yoshitaka Nakamura, Sahoko Ono, Mina Kono, Atsushi Kato, Aki Saito, Eisuke Kondo, Yuhei Tanaka, Hideto Teranishi, Hiroto Akaike, Takaaki Tanaka, Ippei Miyata, Satoko Ogita, Naoki Ohno, Takashi Nakano, Kazunobu Ouchi

**Affiliations:** a Department of Pediatrics, Kawasaki Medical School, Okayama, Japan; b Department of Pediatrics, Aso Iizuka Hospital, Fukuoka, Japan

**Keywords:** antimicrobial susceptibility, children, epidemic, Japan, *Mycoplasma pneumoniae*

## Abstract

We compared the antimicrobial susceptibility of Mycoplasma pneumoniae isolates from pediatric patients in Japan in 2011–2012 and 2015–2016, when epidemics occurred. The antimicrobial activity of macrolides and tetracyclines against M. pneumoniae infection tended to be restored in 2015–2016. There was no change in the antimicrobial activity of quinolones against M. pneumoniae infection.

## Abstract (precis)

We compared the antimicrobial susceptibility of Mycoplasma pneumoniae isolates from pediatric patients in Japan in 2011–2012 and 2015–2016, when epidemics occurred. The antimicrobial activity of macrolides and tetracyclines against M. pneumoniae infection tended to be restored in 2015–2016.

## TEXT

Mycoplasma pneumoniae is an important pathogen that causes human respiratory tract infection, particularly in children and young adults. Epidemics of M. pneumoniae infection occur in 3- to 5-year cycles. In 2011–2012 and 2015–2016 in Japan, the number of patients increased by ∼2-fold the number in a typical year (1).

Macrolides are the first-line treatments for respiratory tract infections caused by M. pneumoniae (2). However, macrolide-resistant (MR) M. pneumoniae isolates were detected in Japanese pediatric patients in 2001 for the first time worldwide and have become widespread in Japan (3). The rate of MR M. pneumoniae infection was as high as 80% among pediatric patients in Japan in 2009 to 2011 (4). We also investigated the prevalence of MR M. pneumoniae since 2008 (5) and reported that the prevalence of MR M. pneumoniae among pediatric patients decreased from 74.6% to 49.5% between 2011 and 2015 in Japan (6).

Tetracyclines or quinolones are recommended for treatment of MR M. pneumoniae infection. Second-line treatments, such as tetracycline and quinolones, are increasingly used because of the increase in MR M. pneumoniae cases in Japan (2).

It is important to conduct surveillance of the susceptibilities of M. pneumoniae isolates to tetracyclines, quinolones, and macrolides. We previously reported that quinolones exhibited potent antimicrobial activity against both MR and macrolide-sensitive (MS) M. pneumoniae isolates from pediatric patients in 2009 to 2011 (7). However, there are no recent reports of antimicrobial activity against M. pneumoniae infection.

We investigated the antimicrobial susceptibility of M. pneumoniae isolates from pediatric patients in Japan in 2011 to 2016 and compared the cumulative distributions of the MICs of macrolides, quinolones, and tetracyclines in 2011–2012 and 2015–2016.

We enrolled all pediatric patients with acute respiratory tract infections at 85 institutions located in 8 areas throughout Japan (20 institutions in Kyushu, 25 in Chugoku, 3 in Shikoku, 11 in Kinki, 7 in Chubu, 3 in Kanto, 2 in Tohoku, and 3 in Hokkaido) in 2011 to 2016.

Pediatricians at the facilities collected samples from patients with suspected M. pneumoniae infections. Informed consent was obtained from the parents of all patients. The Ethics Committee at Kawasaki Medical School, Kurashiki, Japan, approved the study protocol on 15 October 2018 (no. 3119-1).

M. pneumoniae isolates were obtained by cultivation of specimens. The medium used for isolation and determination of the MIC was pleuropneumonia-like organism broth (PPLO) (Oxoid, Hampshire, UK) supplemented with 0.5% glucose (FUJIFILM Wako Pure Chemical Corporation, Osaka, Japan), 20% mycoplasma supplement G (Oxoid), and 0.0025% phenol red (Sigma-Aldrich, St. Louis, MO).

The MICs of antimicrobial agents for the isolated strains were determined with microdilution methods (8). Briefly, medium containing 10^5^ to 10^6^ CFU/ml of M. pneumoniae was added to 96-well microplates and incubated at 37°C for 6 to 8 days.

MIC was defined as the lowest concentration of antimicrobial agent at which the metabolism of the organism was inhibited, which was evidenced by the lack of a color change in the medium 3 days after the drug-free control first showed a color change. The reference strain FH was used as a drug-susceptible control. The antimicrobial agents used for MIC determination were erythromycin, clarithromycin, azithromycin, clindamycin, minocycline, tetracycline, tosufloxacin, garenoxacin, and levofloxacin.

Table 1 shows the MIC range, MIC_50_, and MIC_90_ of the nine antimicrobial agents for 873 MR M. pneumoniae and 383 MS M. pneumoniae isolates.

**TABLE 1:** *In vitro* antimicrobial activity against clinical isolates of Mycoplasma pneumoniae strains

Organism (no. of strains)	Antimicrobial agent^*a*^	MIC (μg/ml)		
		Range	MIC_50_	MIC_90_
Mycoplasma pneumoniae (1,256)	TFLX	0.0625 to 0.5	0.25	0.25
	GRNX	0.0078 to 0.125	0.0313	0.0313
	LVFX	0.25 to 1	0.5	0.5
	TC	0.125 to 1	0.5	0.5
	MINO	0.125 to 4	1	2
	CLDM	0.25 to >128	64	128
	EM	0.001 to >128	>128	>128
	CAM	0.00025 to >128	>128	>128
	AZM	0.0000313 to >128	32	64
Macrolide-susceptible M. pneumoniae (383)	TFLX	0.0625 to 0.5	0.25	0.5
	GRNX	0.0156 to 0.125	0.0313	0.0625
	LVFX	0.25 to 1	0.5	0.5
	TC	0.125 to 1	0.5	0.5
	MINO	0.125 to 4	1	2
	CLDM	0.25 to 4	1	1
	EM	0.001 to 2	0.0039	0.0078
	CAM	0.00025 to 0.5	0.002	0.0039
	AZM	0.0000313 to 0.0313	0.00025	0.0005
Macrolide-resistant M. pneumoniae (873)	TFLX	0.0625 to 0.5	0.25	0.25
	GRNX	0.0078 to 0.063	0.0313	0.0313
	LVFX	0.25 to 1	0.5	0.5
	TC	0.125 to 1	0.5	0.5
	MINO	0.125 to 4	1	2
	CLDM	4 to >128	128	128
	EM	32 to >128	>128	>128
	CAM	8 to >128	>128	>128
	AZM	0.25 to >128	32	64

a TFLX, tosufloxacin; GRNX, garenoxacin; LVFX, levofloxacin; TC, tetracycline; MINO, minocycline; EM, erythromycin; CAM, clarithromycin; AZM, azithromycin.

The Japanese Society for Mycoplasmology has proposed resistance breakpoints for the compounds used against M. pneumoniae isolates (9). The criteria for drug-resistant M. pneumoniae are MICs of ≥16 μg/ml for erythromycin, clarithromycin, and azithromycin. The MIC_50_ and MIC_90_ of the macrolides erythromycin, clarithromycin, and azithromycin for the isolates were >128/>128, >128/>128, and 32/64 μg/ml, respectively. The MIC_50_/MIC_90_ values of macrolides for MS M. pneumoniae isolates were lower than those of the other antimicrobials. All quinolones, particularly garenoxacin, showed potent antimicrobial activity against MR M. pneumoniae, with MIC_50_/MIC_90_ values of 0.0313/0.0313 μg/ml. These values were equal to those of MS M. pneumoniae isolates. Tosufloxacin, the only quinolone approved for treatment of pneumonia in pediatric patients in Japan, also showed good activity against MR and MS M. pneumoniae isolates, with MIC_50_s/MIC_90_ values of 0.25/0.25 and 0.25/0.5 μg/ml, respectively. Tetracyclines, such as tetracycline and minocycline, showed comparably good activity against MR and MS M. pneumoniae isolates.

**FIG 1:**
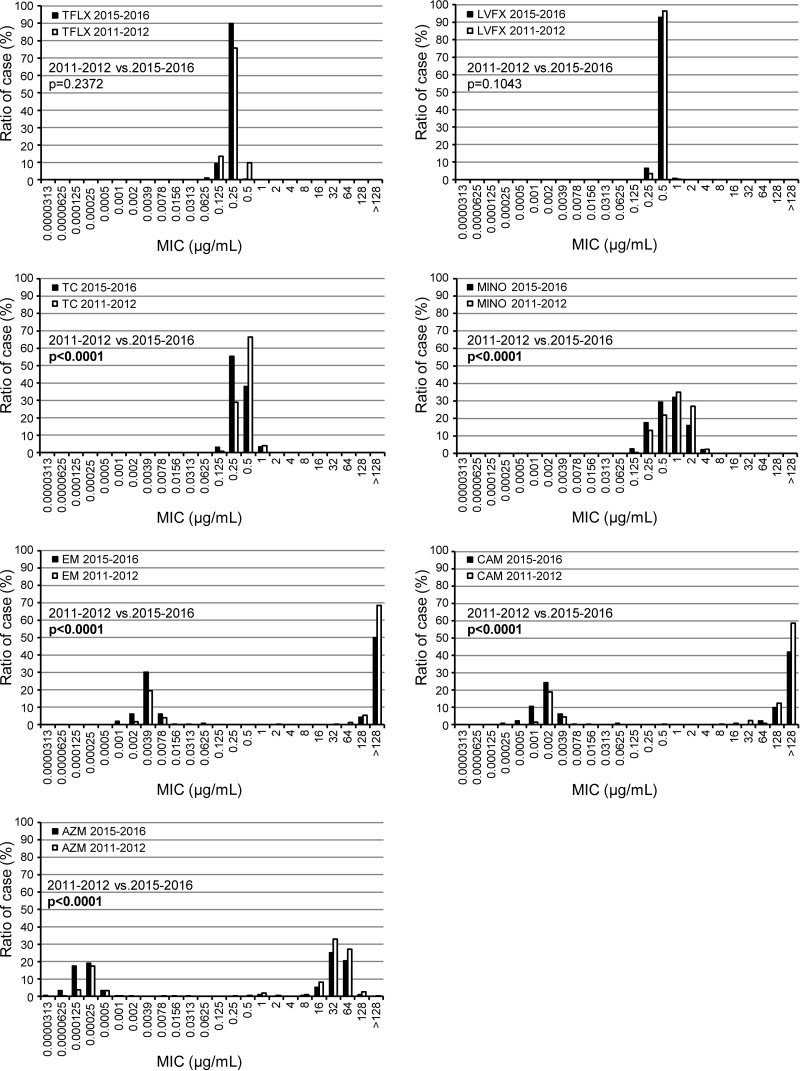
MIC distribution of antimicrobial agents for M. pneumoniae isolates in two recent epidemics in 2011–2012 and 2015–2016. TFLX, tosufloxacin; LVFX, levofloxacin; TC, tetracycline; MINO, minocycline; EM, erythromycin; CAM, clarithromycin; AZM, azithromycin.

Figure 1 shows the MIC distribution of macrolides, quinolones, and tetracyclines in 2011–2012 and 2015–2016 and statistical analysis of the differences in each MIC value between the two periods by the Wilcoxon rank-sum test. The resistance rate of erythromycin, clarithromycin, and azithromycin decreased from 75%, 74%, and 71.9% in 2011–2012 to 54.2%, 54.2%, and 53.1% in 2015–2016, respectively. The MIC values of macrolides and tetracyclines in 2015–2016 were significantly lower than those in 2011–2012. The antimicrobial activity of quinolones remained potent in 2016. Strains resistant to these agents were not detected in this study.

In comparing the two periods when M. pneumoniae epidemics occurred (2011–2012 and 2015–2016), the antimicrobial activities of all macrolides and tetracyclines against M. pneumoniae isolates were restored significantly in 2015–2016. The sensitivity to macrolides may have been restored because of a decrease in M. pneumoniae isolates with specific point mutations in domain V of the 23S rRNA gene (6).

We considered two reasons for recovery of the sensitivity to macrolides. One is the appropriate use of tosufloxacin for treating M. pneumoniae infection, and the other is a shift in the P1 type.

First, tosufloxacin was approved in 2010 in Japan as treatment for pediatric patients and is recommended for use in patients with suspected MR M. pneumoniae infection as a second-line drug under various guidelines (2). Specifically, tosufloxacin is recommended for cases with M. pneumoniae infection in which fevers are not reduced by 48 to 72 h after the initiation of macrolide treatment. Ouchi et al. (10) reported that tosufloxacin was significantly more effective than clarithromycin in eradicating MR M. pneumoniae. Additionally, total oral antimicrobial use of macrolides decreased, whereas that of quinolones, including tosufloxacin, increased from 2011 to 2013 in children (age, 0 to 14 years), based on analysis of health insurance claim data in the national database (11). Miyashita et al. (12) reported lower macrolide resistance rates of M. pneumoniae infection in adults to whom macrolides, tetracyclines, or respiratory quinolones were commonly administered than in children to whom only macrolides or tetracyclines were administered in 2008 to 2011. Thus, because tosufloxacin was used appropriately for M. pneumoniae infections, the development of MR M. pneumoniae was prevented.

Second, a type shift in *p1* may explain the recovery of sensitivity to macrolides.

At the surface of the attachment organelle is the 170-kDa adhesin protein P1, which is densely clustered and plays a major role in binding to the receptor molecule of host epithelial cells (13). Two major subtypes of *p1* (subtypes 1 and 2) are known that form some minor variants (subtype 1, 2a, 2b, and 2c).

A type-shift phenomenon occurs in Japan every 8 to 10 years. A major subtype of *p1* was subtype 2 in 1995 to 2001. Thereafter, subtype 1 reached a level of 90% in 2005, whereas subtype 2 decreased from 2001 to 2005. Recently, it was reported that a type shift from subtype 1 to subtype 2 occurred in 2013 to 2015 in Yamagata Prefecture, Japan (14). It was presumed that because this subtype had few opportunities to be exposed to macrolides since 2000, isolates of subtype 2 may have been more sensitive to macrolides than isolates of subtype 1. Furthermore, correlations of P1 with multilocus variable-number tandem-repeat analysis (MLVA), which is one of the methods for typing, have been described (15, 16). As revealed by a previous MLVA-4 analysis, almost all isolates of 4/5/7/2 or 4/5/7/3 strains belonged to subtype 1 of *p1*, whereas almost all of the 3/5/6/2 or 3/6/6/2 strains belonged to subtype 2 of *p1*. We did not perform MLVA, and we hope to address this aspect in the future.

Next, we discuss the reason that the MIC values of tetracyclines against M. pneumoniae were restored significantly in 2015–2016 compared with 2011–2012. Okubo et al. (17) investigated the trends of use in practice patterns on pediatric M. pneumoniae-related respiratory infections. They reported that the usage of tetracyclines against pediatric M. pneumoniae-related respiratory infections decreased after the pandemic of M. pneumoniae infections in 2011–2012. Although they did not investigate the cases in 2015–2016, we suggest that the use of tetracycline in 2015–2016 might not have increased as much as in 2011–2015 because of the recommendation of quinolones against pediatric M. pneumoniae infections. In other words, because quinolones were not recommended in 2011–2012, some cases of children (<8 years old) suspected to have MR M. pneumoniae infections were prescribed tetracyclines. If quinolones were prescribed instead of tetracyclines in these cases in 2015–2016, the chances of prescribing tetracyclines may have decreased.

In summary, quinolones and tetracyclines exhibited potent antimicrobial activities against MS and MR M. pneumoniae infection in 2011–2012 and 2015–2016, when M. pneumoniae epidemics occurred. The antimicrobial activities of macrolides and tetracyclines were restored significantly in 2015–2016 compared with 2011–2012.

The antimicrobial susceptibility of M. pneumoniae isolates should continue to be surveyed in Japan and other countries.
